# First Record of the Entomopathogenic Nematode *Steinernema litorale* (Filipjev) (Rhabditida: Steinernematidae) and Its Symbiotic Bacterium from Turkey, and Its Efficacy Capability

**DOI:** 10.3390/insects11030144

**Published:** 2020-02-25

**Authors:** Esengül Özdemir, Şerife Bayram, İ. Alper Susurluk

**Affiliations:** 1 Department of Plant Protection, Faculty of Agriculture, Ankara University, Diskapi, 06110 Ankara, Turkeybayram@agri.ankara.edu.tr (Ş.B.); 2 Department of Plant Protection, Faculty of Agriculture, Bursa Uludağ University, Nilüfer, 16059 Bursa, Turkey

**Keywords:** entomopathogenic nematode, *Steinernema litorale*, *Xenorhabdus bovienii*, Turkey

## Abstract

The entomopathogenic nematode *Steinernema litorale* was isolated from Çamkoru Nature Park located in Ankara, Turkey, in September 2018. *Steinernema litorale* was recovered in 1 of 67 soil samples from a natural forest area; the soil was characterised as sandy loam. The isolated nematode *S. litorale* was identified based on morphological and molecular parameters. The symbiotic bacterium of *S. litorale* was determined as *Xenorhabdus bovienii*. *Steinernema litorale* was found for the first time in Turkey and the Middle East. The virulence of the isolate was tested on *Galleria mellonella* larvae. Different concentrations of the nematode (10, 25, 50, 75, and 100 infective juveniles (IJs/larvae) were used. While the LC_50_ values at 48 h, 72 h, and 96 h were 153.419, 51.005, and 15.439 IJs, respectively, and the LT_50_ values at 75 IJs and 100 IJs showed that this isolate is capable to control insect larvae within 50.083 and 36.266 h, respectively.

## 1. Introduction

Entomopathogenic nematodes (EPNs) have great potential as biocontrol agents for both soil dwelling and above-ground insect pests [1,2]. EPNs are symbiotically associated with the bacteria genus *Xenorhabdus* in steinernematids and *Photorhabdus* in heterorhabditids [3]. These bacteria are lethal to their insect host, which makes them suitable for using as biocontrol agents in integrated pest management programs throughout the world [4]. The effective use of EPNs as a biopesticide, therefore, depends on the isolation of native species, as they are adapted to the prevalent environment and climatic conditions of that particular area, as well as on the proper identification of these species [5,6].

At present, there are 95 steinernematid and 16 heterorhabditid species determined worldwide [7], with the exception of Antarctica [8]. It has been more common and frequent to recover steinernematids than heterorhabditids throughout the world. This proves that steinernematids appear to be more widely distributed than heterorhabditids in the soil [8]. In Turkey, several surveys have been conducted to isolate the indigenous EPNs, one of which resulted in the discovery of a new *Steinernema anatoliense* [9] from Eastern Anatolia, Turkey. There are a total of 12 species of valid entomopathogenic nematodes documented from Turkey [10,11,12,13,14,15,16], and these species include *Heterorhabditis bacteriophora*. *H. indica*, *H. marelatus*, *H. megidis*, *S. affine*, *S. anatoliense*, *S. bicornutum*, *S. carpocapsae*, *S. feltiae*, *S. weiseri*, *S. websteri*, and *S. krausseri*. 

Until the recent past, an entomopathogenic nematode description was based primarily on the overlap of morphometry and related morphology, but this method is not easily applicable for their identification [8,17]. On the other hand, the application of molecular methods, such as polymerase chain reaction (PCR) and sequence analysis, in order to deal with taxonomic ambiguities and help resolving problems such as identification of organisms that are morphologically similar, are considered as complementary to traditional identification methods [18].

During the survey of EPNs in the non-agricultural fields of Ankara, Turkey, a nematode isolate belonging to *Steinernema*, Travassos, 1927, was recovered from the soil samples of Çamkoru Nature Park. Morphological, morphometric, and molecular studies showed that this nematode isolate is conspecific to *Steinernema litorale***,** Yoshida, 2004 (Rhabditida: Steinernematidae), with slightly larger infective juveniles (IJs). Furthermore, we tested virulence of this nematode and performed a molecular identification of its bacterial symbiont. The current study reports a new species of EPN, *S. litorale*, and its symbiotic bacterium *Xenorhabdus bovienii* from Turkey and the Middle East. 

## 2. Materials and Methods 

### 2.1. Origin and Isolation of the Nematode

In total, 67 soil samples were collected from Çamkoru Nature Park, Ankara. Sampling details are indicated in Table 1. The park is located in a transition zone between continental and temperate climates and it is a *Pinus nigra* Arn.-dominated nature forest. The *Galleria*-larvae baiting technique was used to recover the nematodes [19]. The insect cadavers were surface sterilized in a 0.1% NaOCl solution, washed in distilled water, and transferred onto a White trap [20]. Infective juveniles were applied to the last instars of *Galleria mellonella* L. (Lepidoptera: Pyralidae) larvae in order to confirm the pathogenicity of the isolate. The isolated IJs were washed three times with distilled water, and stored in a tissue culture flask at 12 °C. These surface-sterilised nematodes were used for molecular and morphological identification.

### 2.2. Morphology and Morphometry 

The male and infective juvenile (IJ) stages of the isolate were obtained from *G. mellonella* cadavers. For the identification of the isolate, 20 specimens from each developmental stage were randomly taken from the cadavers. The collected nematodes were first taken into the Ringer’s solution and then they were left in a 60 °C water bath for 2 min and they were allowed to die without being exposed to osmotic stress. The dead nematodes fixed and were transferred to anhydrous glycerine according to [21], and mounted into a drop of glycerin on slides. Morphological observations and morphometric measurements were made using a Leica DM4500 light microscope. 

### 2.3. Cross-Hybridization

Cross-breeding tests were performed between *S. litorale* and morphologically similar Turkish isolates *S. feltiae* and *S. affine* using the modified hanging-drop method, as described by Kaya and Stock (1997) [22]. Twenty replicates were observed for each treatment and control. Sealed Petri dishes were incubated at 25 °C and observations were recorded daily for reproductive compatibilities.

### 2.4. Molecular Identification of the Nematode

Total genomic DNA was extracted from freshly harvested nematodes by using a Genematrix Tissue and Bacterial DNA Purification Kit following by manufacturer’s instructions. PCR amplification and sequencing of the ITS region were used to identify and determine the phylogenetic relationship between the isolates. The ITS regions of the rDNA were amplified using forward TW81 5′-GTTTCCGTAGGTGAACCTGC-3′ and reverse AB28 5′-ATATGCTTAAGTTCAGCGGGT-3′ [23]. The cycling parameters included denaturation at 94 °C for 4 min, followed by 35 cycles of 94 °C for 1 min, 58 °C for 1 min, and 72 °C for 2 min, followed by a post-amplification extension at 72 °C for 10 min. 

The presence of DNA fragments and yield were measured by agarose gel electrophoresis (1.5% in 1X TBE buffer). PCR products were directly sequenced in both directions at BM Labosis (Turkey). Chromas 2.6.6 was used for editing the generated sequence. Afterwards, it was compared with the sequences present in the National Centre for Biotechnology (NCBI) by means of a BLAST search. A cut of ≥99% identity was considered for the same species. Phylogenetic analysis was performed using the avaliable representative species of the genus *Steinernema* obtained from the NCBI’s GenBank database. Sequences were aligned using Clustal-W on MEGAX before generating the phylogenetic tree [24]. The phylogenetic tree was generated using the maximum likelihood (ML) method based on the Tamura-Nei model.

### 2.5. Isolation and Molecular Characterization of the Bacterium 

The symbiotic bacteria were obtained from the hemolymph of *G. mellonella* 1 d after infection with *S. litorale* CTP19-1 following Akhurst’s (1980) [25] methodology. The hemolymph was streaked on NBTA (nutrient agar supplemented with 0.004% (*w*/*v*) triphenyltetrazolium chloride and 0.0025% (*w*/*v*) bromothymol blue) and left overnight at 28 °C. Single colonies were transferred with a sterile loop to YS broth and cultivated on an orbital shaker (180 rpm) at 25 °C

The genomic DNA of the symbiotic bacterium was isolated from bacterial pellets by using the Genematrix Tissue and Bacterial DNA Purification Kit following the manufacturer’s instructions and stored at −20 °C for further use in PCR studies. 

For amplification of 16S rDNA, 16S_F 5′-GAAGAGTTTGATCATGGCTC-3′ and 16S_R 5′-AAGGAGGTGATCCAGCCGCA-3′ [26] primers were used. PCR cycling parameters were an initial step of 94 °C for 5 min, followed by 30 cycles of 94 °C for 1 min, 50 °C for 1 min, 72 °C for 2 min, and a final extension of 72 °C for 7 min. PCR products were visualized by using agarose-gel electrophoresis. PCR products were directly sequenced by BM Labosis (Turkey). Chromas 2.6.6 was used for editing the sequence generated. Afterwards, it was compared with the sequences present in the National Centre for Biotechnology (NCBI) by means of a BLAST search. A cut of ≥99% identity was considered for the same species. Phylogenetic analysis was performed using the avaliable representative species of the genus *Xenorhabdus* obtained from the NCBI’s GenBank database. Sequences were aligned using Clustal-W on MEGAX before generating the phylogenetic trees [24]. The phylogenetic tree was generated using the maximum likelihood (ML) method based on the Hasegawa-Kishino-Yano model.

### 2.6. Insecticidal Activity

In order to determine the efficacy of *S. litorale* (CTP19-1) on *G. mellonella*, 10 g of sterilized sand was added into each well of the 24-well plates. Then the nematodes were applied onto the sand at 10, 25, 50, 75, and 100 IJs per larvae. Just distilled water was applied to the control plates. The treated well-plates were kept at room temperature for 1 h, and then a single last instar *G. mellonella* larva was placed on the sand surface and the plates were capped with a lid. 24, 48, 72, and 96 h after the application, each plate was controlled and the number of dead larvae was recorded. The experiment was repeated 2 times on different days under the same conditions. The nematode infection was confirmed by dissection of dead larvae under a stereomicroscope. 

### 2.7. Statistical Analysis 

The mortalities in the control and in the treatment groups were determined at 24, 48, 72, and 96 h after the nematode exposure. The mortality rates in the treatment groups were adjusted against the mortality in the control [27]. To determine differences among different concentrations, the data were subjected to analysis of variance and subsequently to a Tukey multiple comparison test. This analysis was performed using IBM SPSS 22.0. LC_50_ values were calculated using Polo Plus 1.

## 3. Results

Results obtained through morphology, morphometry, cross-hybridization, and molecular analyses identified the isolated nematode (CTP19-1) as *S. litorale* [28]. 16S rDNA sequence and phylogenetic analyses showed that *S. litorale* CTP19-1 is symbiotically associated with the bacterium *X. bovienii*.

### 3.1. Morphology and Morphometry

All morphological examination results of the IJs and males are shown in Table 2. Morphological traits of the examined specimens for the isolate matched the original description of the species. The morphological features of the IJs and males of the isolate from the genus *Steinernema* resemble the original description of *S. litorale* by [28]. The population of the Turkish strain has some slight variations viz. the IJs’ body length of 909 µm (834–988) of the original description of *S. litorale* is shorter than for this isolate, which has a body length (L) of 919.0 µm (850.3–991.5); the mucron length of 3.6 ± 3.2 (0–8.1) and tail length of 81.8 ± 4.2 (69.8–90.2) of this isolate are a little shorter the original isolate [28]. However, the maximum body width (W), distance from anterior end to nerve ring (NR), distance from anterior end to base of basal bulb (ES), anal body width (ABW), and %E for both IJs and the males of our isolate and the original discription are similar. The length of the male’s spicula and gubernaculum are also within the range in the original description (Table 2). 

### 3.2. Cross-Hybridization

Progeny were not observed among cross-breeding treatments. Progeny were produced in the self-cross controls.

### 3.3. Molecular Identification and Phylogenetic Analysis of Nematode Isolate

**Figure 1:**
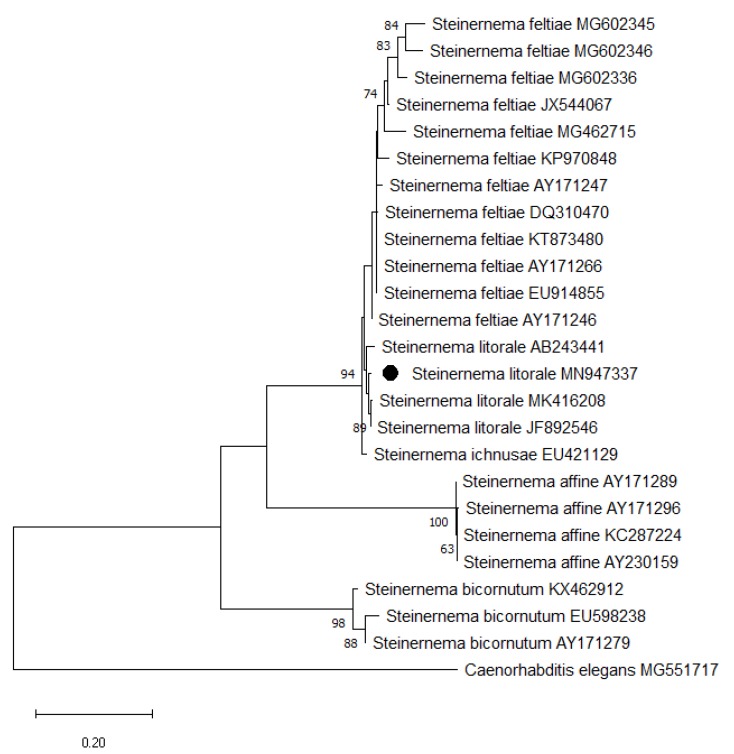
Phylogenetic relationships of the *Steinernema* species based on analysis of ITS rDNA regions. The tree was reconstructed by the maximum-likelihood method. Sequences of *Caenorhabditis elegans* (MG551717) was used as the outgroup species. The GenBank accession numbers are indicated after the name of the bacterial isolates. The number on the branches 60% indicates the percentage of 1000 bootstrap replicates. The bar represents 0.02 substitutions per site.

The generated sequence of the nematode isolate *S. litorale* CTP19-1 (MN947337) was 970 bp long. A BLAST search of the ITS rDNA region of the isolate indicated a 99.79% similarity with *S. litorale* (MK416208) and 98.65% and 97.01% similarity with the sequences of the two *S. litorale* strains previously isolated and submitted to GenBank (accession numbers JF892546 and AB243441). Other sequence comparisons also indicated a 97.01% and 96.90% similarity with two *Steinernema* spp. (AY230167 and GU395624). The new isolate classified in a clade with the other isolates of the Steinernema species as a result of the phylogenetic analysis (Figure 1).

### 3.4. Molecular Identification and Phylogenetic Analyses of Bacterium

**Figure 2:**
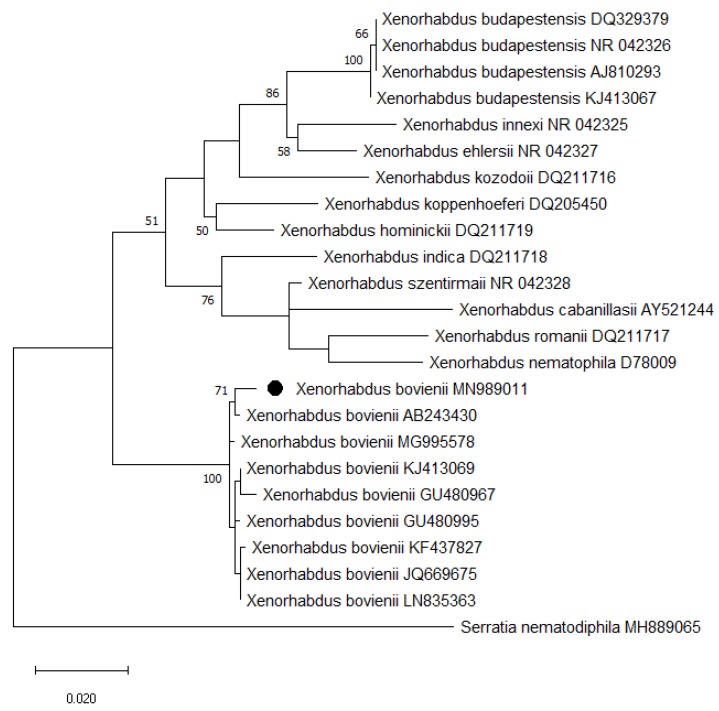
Phylogenetic relationship of representative *Xenorhabdus* bacteria associated with steinernematids based on the 16S rRNA gene sequences. The tree was reconstructed by the maximum likelihood method. *Xenorhabdus bovienii* isolated from *S. litorale* CTP19-1 is shown as labeled. The 16S rRNA gene sequences of *Serratia nematodiphila* (MH889065) was used as the outgroup species. The GenBank accession numbers are indicated after the name of bacterial isolates. Numbers more than 50% at branch-point indicate in percentage how often the corresponding cluster was found among the 1000 intermediate trees. The bar represents 0.02 substitutions per site.

The generated sequence of *Xenorhabdus bovienii* (MN989011) is 1381 bp long and has 99.71% similarity with the *X. bovienii* (MH595982) isolate. The evolutionary history was inferred by using the maximum likelihood method and Hasegawa-Kishino-Yano model. Based on the sequence analyses of the 16S rDNA gene, a highly supported group of the *Xenorhabdus bovienii* CTP19-1 isolate and *X. bovienii* AB243430 is shown (Figure 2). So far, *X. bovienii* AB243430 was isolated from the first identified *S. litorale* AiAt199 isolate [29].

### 3.5. Insecticidal Activity 

Analysis of variance revealed significant differences among exposure times and varied concentrations of *S. litorale* CTP19-1 on *G. mellonella* mortality (*p* < 0.005).

**Figure 3:**
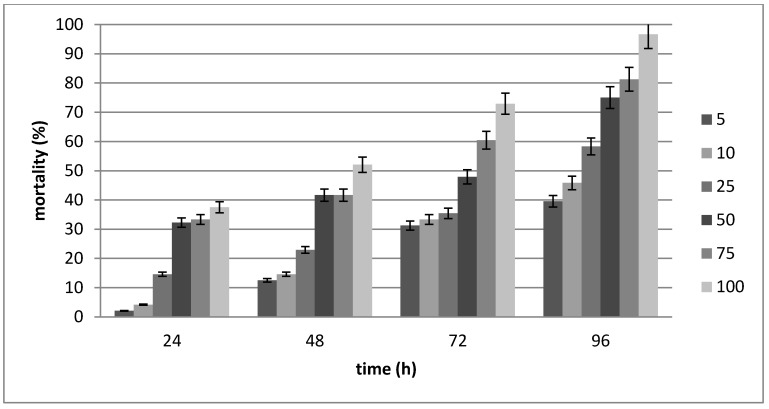
Percentage of mortality of *G. mellonella* larvae with different doses of *S. litorale* CTP19-1.

In the dose response tests of *S. litorale* CTP19-1, *G. mellonella* mortality reached 96.67% within 96 h at 100 IJs concentration. The results show that the concentration of IJs affected the mortality of *G. mellonella* larvae differently (*p* < 0.05) (Figure 3, Table 3). The LC_50_ at 48 h, 72 h, and 96 h showed that CTP19-1 was capable of killing the *G. mellonella* larvae with 153.419, 51.005, and 16.439 IJs, respectively (Table 4). The LT_50_ at 75 IJs and 100 IJs showed that this isolate is capable of controlling *G. mellonella* larvae within 50.083 and 36.266 h, respectively (Table 5). 

## 4. Discussion

The entomopathogenic nematodes from the genera *Steinernema* and *Heterorhabditis* are used to control a number of agricultural pests worldwide [8]. When EPNs are applied under suitable conditions, their effectiveness is considered to be equal to chemical pesticides [30,31]. The effective use of EPNs as biopesticides, therefore, depends on the isolation of native species, as they are adapted to the prevalent environment and climatic conditions of that particular area, as well as the accurate identification of these species [5,6,32]. However, diagnosis of EPNs to the species level based on traditional methods may cause problems [33]. Therefore, the main aim of this study was to identify the isolate *S. litorale* in combination of morphometric and molecular data. Another aim of the study was to determine whether this identified *S. litorale* isolate has potential to be used as an effective biological control agent in the future. Finally, the bacterium *Xenorhabdus bovienii*, which has a symbiotic relationship with this isolate, was also defined molecularly [34]. 

The morphometric measurements have shown that the *Steinernema litorale* CTP19-1 isolate has a negligible variation within the range of *Steinernema litorale* described by [28]. These differences are thought to result from both intraspecific genetic variations and environmental factors. *Steinernema litorale* has been discovered in Japan [28] and following this discovery it has been obtained only in Pakistan [35] and China [36] and never recorded elsewhere. At this point it is important to mention that an isolate recovered from Italy, which was thought to be *S. litorale* according to RFLP types by Susurluk et al. (2007) [34], was later on determined to be *S. ichnusae* with DNA analyses of the internal transcribed spacers and D2D3 regions [37]. The phylogenetic tree presented in this study has also proved that these two entomopathogenic nematode species are closely related.

In the present study, the *Steinernema litorale* CTP19-1 isolate was recovered from a natural forest area (Çamkoru Nature Park) in Central Anatolia, Turkey. The relationship between habitat features and abundance of EPNs has been stated in several studies. It has been reported that there are high occurrence rates of EPNs in disturbed habitats, such as agricultural areas with high anthropogenic influences [38,39,40]. However, in undisturbed habitats, abundance of EPNs is relatively higher [5,41,42,43]. From this point of view, it can be said that EPN occurrence varies among species depending on habitat types and geographical regions.

The phylogenetic analysis inferred from the ITS rDNA region indicated that *Steinernema litorale* is a species forming a monophyletic group with *S. feltiae*, *S. affine*, and *S. ichnusae*. The interaction between the species in the steinernematids and their symbiotic bacteria is species-specific; except for a few numbers of *Xenorhabdus* species [44]. The symbiotic bacteria associated with *Steinernema litorale* is *Xenorhabdus bovienii* [29]. For identification and phylogenetic analysis, the sequence of the 16S rRNA gene is widely used. On the other hand, although the phylogenetic information of the 16S rRNA sequence is insufficient due to strict functional limitations, this region still maintains its importance in bacterial identification studies [45,46]. We also used 16S rRNA gene for identification of the bacterium symbiotically associated with the Turkish *S. litorale* isolate, and determined it as *X. bovienii*. 

In Turkey, several research activities were undertaken on EPNs and are still going on. However, the country constitutes a fertile field, promising for EPN exploration, since for the few cities surveyed, only a small number of EPNs have been found, including one new species reported. So far, little is known about nematology in general in Turkey and about EPNs in particular. In the country, to our knowledge, eight species of *Steinernema* and four species of *Heterorhabditis* have been found in several provinces, including *Steinernema affine*, *S*. *anatoliense, S. bicornutum, S*. *carpocapse, S*. *feltiae*, *S*. *weiseri*, *S. websteri, S. krausseri*, *H*. *bacteiophora*, *H. indica*, *H*. *megidis,* and *H*. *marelatus* [10,11,12,13,14,15,16]. In Turkey, two species of *Xenorhabdus* also have been reported, including *X*. *nematophila* and *X*. *bovienii.* Additionally, 2 species with 2 subspecies of *Photorhabdus*, including *Photorhabdus kayaii*, *P*. *luminescens* subsp. *kayaii*, and *Photorhabdus thracensis* [43,45]. Most surveyed locations for EPNs and their symbiotic bacteria were field crops and fruit crops, with a minor amount of the researches being roadsides in Turkey. Although Ankara is located in the Central Anatolia region, it is a city with an occurrence of different climatic influences. Especially, most of the Nature Parks of Ankara are located in climatic transition zones. Çamkoru Nature Park is one of them, and we think that it is not a coincidence to recover a new entomopathogenic nematode record for the country at this location.

## 5. Conclusions

*Steinernema litorale* was first isolated from soil samples in Japan [28]. Since then, *S. litorale* has also been reported in soil samples from China and Pakistan [35,36]. The findings of this study confirm the previous studies showing the extent of this species in the northern hemisphere. In conclusion, this is the first record of *Steinernema litorale* and its symbiotic bacteria *Xenorhabdus bovienii* in both Turkey and also in the Middle East. In addition, the biological activity of this isolate has been tested. In this context, it is of great importance to determine the national distribution of *S. litorale*, as we have recorded it for the first time in Turkey, in both undisturbed and disturbed areas. Besides, determining the efficacies of the identified *S. litorale* isolates on important agricultural pests will contribute to the use of these isolates in local IPM programs.

## Figures and Tables

**Table 1:** Locality, habitat, sampling date, and soil characteristics of the positive sample.

Sampling				Soil Characteristics			
Location	Coordinates	Sampling Date	Habitat	Soil Type	pH	Moisture (%)	Temperature (°C)
Çamkoru Nature Park, Ankara	40°60′380′′ N 32°47′4581′′ E	September 2018	Pine Forest	Sandy loam	5.9	14	13

**Table 2:** Morphometrics of *Steinernema litorale* CTP19-1. All measurements are in micrometers (except n, ratio, and percentage). Data are expressed as mean ± SD (range).

Characters	*Steinernema litorale* CTP19-1		*Steinernema litorale* n. sp. (Yoshida, 2004)	
	Infective Juvenile	1st Generation (Male)	Infective Juvenile	1st Generation (Male)
n	20	20	25	25
L	919.0 ± 44.2 (850.3−991.5)	1346.0 ± 96.2 (1221.3−1498.7)	909 ± 42 (834−988)	1360 ± 84 (1230−1514)
EP	61.7 ± 5.1 (55.2−68.6)	92.3 ± 9.1 (73−104)	61 ± 3.3 (54−69)	96 ± 7.0 (76.6−107)
NR	97.6 ± 5.3 (91.1−106.9)	111 ± 8.5 (90−123)	96 ± 4.0 (89−104)	114 ± 10.8 (94−128)
ES	124.9 ± 4.5 (117.3−131.5)	139.2 ± 8.9 (126.2−157.5)	125 ± 4.6 (114−133)	(133−163) 34 ± 3.7
T	81.8 ± 4.2 (69.8−90.2)	31 ± 6.5 (22−40)	83 ± 4.5 (72−91)	34 ± 3.7 (26−41)
ABW	19.8 ± 1.1 (16.4−23.0)	44.1 ± 3.6 (37.6−49.5)	19 ± 1.5 (16−22)	43 ± 3.0 (37−49)
W	31.8 ± 2.8 (28.2−33.6)	95.4 ± 7.3 (79.2−106.8)	31 ± 1.4 (28−33)	96 ± 7.9 (82−111)
SL	NA	72 ± 5.3 (64−88)	NA	75 ± 4.8 (67−89)
GL	NA	51 ± 4.8 (42−63)	NA	53 ± 4.0 (44−64)
Mucro	NA	3.6 ± 3.2 (0−8.1)	NA	3.9 ± 2.1 (0−8.5)
a	30.0 ± 1.6 (27.2−32.2)	14 ± 1.1 (12−16)	29.5 ± 0.8 (27.2−30.9)	14.3 ± 0.9 (12.3−16.1)
b	7.2 ± 0.4 (6.6−8.0)	9.1 ± 0.8 (8.1−10.6)	7.3 ± 0.3 (6.7−7.9)	9.3 ± 0.5 (8.3−10.2)
c	10.7 ± 1.2 (9.5−12.1)	39.8 ± 5.4 (32.1−54.5)	11.0 ± 0.5 (9.7−11.9)	40.7 ± 5.6 (33.2−55.7)
E%	0.76 ± 0.02 (0.71−0.83)	2.63 ± 0.29 (2.28−3.73)	0.73 ± 0.05 (0.68−0.84)	2.86 ± 0.40 (2.31−3.82)
D%	50 ± 3.6 (46.2−57)	66.6 ± 4.9 (55.8−73.1)	48.5 ± 2.9 (44.0−56.0)	65.2 ± 4.4 (55.0−72.4)

NA = not available, n = number of specimens, L = total body length, EP = distance from anterior end to base excretory pore, NR = distance from anterior end to nerve ring, ES = distance from anterior end to base of basal bulb, T = tail length, ABW = anal body width, W = maximum body width, SL = spicule length, GL = gubernaculum length, GW = gubernaculum width, V% = (distance from anterior end to vulva/total body length) × 100, a = L/W, b = L/ES, c = L/T, D% = (EP/ES) × 100, and E% = (EP/T) × 10.

**Table 3:** Mortality rates (%) of *G. mellonella* treated with *S. litorale* CTP19-1.

Species	Time	Concentration (IJs)					
		5	10	25	50	75	100
***S. litorale* CTP19-1**	**24**	2.08 ± 4.17 Cc ^1^	4.17 ± 4.81 Cb	14.58 ± 7.98 BCc	32.25 ± 7.98 ABb	33.33 ± 11.79 ABc	37.5 ± 10.76 Ac
	**48**	12.50 ± 4.81 Bbc	14.58 ± 7.98 Bb	22.92 ± 7.98 Bbc	41.67 ± 6.80 Ab	41,67 ± 11.79 Abc	52.08 ± 7.98 Ac
	**72**	31.25 ± 7.98 Cab	33.33 ± 6.80 Ca	35.42 ± 12.50 Cb	47.92 ± 10.49 BCb	60,42 ± 14.13 ABb	72.92 ± 4.17 Ab
	**96**	39.58 ± 10.49 Ea	45.83 ± 8.33 DEa	58.33 ± 6.80 CDa	75.00 ± 6.80 BCa	81.25 ± 4.17 ABa	96,67 ± 7.98 Aa

^1^ Capital letters among times, and lowercase letters among doses in each time are indicated as statistically different from each other (*p* < 0.05, Tukey test).

**Table 4:** Dose effect of *S. litorale* CTP19-1 species on *G. mellonella*.

Time ^a^	DF	Slope ± SEMc	LC_50_ (95% CL ^b^)
24 h	10	1.430 ± 0.333	286.711 (157.383−1273.741)
48 h	10	1.126 ± 0.210	153.419 (95.494−376.130)
72 h	10	1.135 ± 0.179	51.005 (37.201−77.021)
96 h	10	1.313 ± 0.177	15.439 (10.844−20.458)

^a^ Application times. ^b^ Confidence limits, CL, are given in parentheses.

**Table 5:** Time effect of *S. litorale* CTP19-1 on *G. mellonella*.

Concentration ^a^	DF	Slope ± SEMc	LT_50_ (95% CL ^b^)
5 IJs	6	3.454 ± 0.962	135.261 (99.177−319.460)
10 IJs	6	3.305 ± 0.722	106.282 (84.709−169.850)
25 IJs	6	2.725 ± 0.524	89.332 (65.854−115.172)
50 IJs	6	2.656 ± 0.484	74.343 (54.398−87.564)
75 IJs	6	2.648 ± 0.451	50.083 (36.322−55.622)
100 IJs	6	3.118 ± 0.463	36.266 (25.281−43.056)

^a^ Application doses. ^b^ Confidence limits, CL, are given in parentheses.
